# Neurodevelopment regulators miR-137 and miR-34 family as biomarkers for early and adult onset schizophrenia

**DOI:** 10.1038/s41537-021-00164-1

**Published:** 2021-07-05

**Authors:** Bao-Yu Chen, Jin-Jia Lin, Ming-Kun Lu, Hung-Pin Tan, Fong-Lin Jang, Sheng-Hsiang Lin

**Affiliations:** 1 Institute of Clinical Medicine, College of Medicine, National Cheng Kung University, Tainan, Taiwan; 2 Department of Psychiatry, Chi Mei Medical Center, Tainan, Taiwan; 3 Department of Health, Jianan Mental Hospital, Tainan, Taiwan; 4 Department of Applied Life Science and Health, Chia Nan University of Pharmacy and Science, Tainan, Taiwan; 5 Department of Environmental and Occupational Health, College of Medicine, National Cheng Kung University, Tainan, Taiwan; 6 Department of Public Health, College of Medicine, National Cheng Kung University, Tainan, Taiwan; 7 Biostatistics Consulting Center, National Cheng Kung University Hospital, College of Medicine, National Cheng Kung University, Tainan, Taiwan

**Keywords:** Biomarkers, Schizophrenia

## Abstract

Early-onset schizophrenia (EOS) may have stronger familial aggregation and a more severe outcome than adult-onset schizophrenia (AOS). MicroRNA (miRNA) takes on dual roles as a genetic and epigenetic modulator, which may mediate the influence of genetic risk. Neurological soft signs (NSS) are neurological abnormalities that may be intermediate phenotypes or endophenotypes for schizophrenia. Our previous study found poorer performance on NSS tests from patients with EOS and their unaffected first-degree relatives. Thus, we aimed to identify a set of aberrant neurodevelopmental-related miRNAs that could serve as potential biomarkers for EOS or schizophrenia with NSS. This study included 215 schizophrenia patients (104 EOS and 111 AOS), 72 unaffected first-degree relatives, 31 patients with bipolar disorder, and 100 healthy controls. Differential expression analysis revealed that miR-137, miR-34b, and miR-34c were significantly up-regulated in patients with schizophrenia and their unaffected first-degree relatives compared to healthy controls. Receiver operating characteristic (ROC) analysis showed that the miR-137 expression signature could be used to discriminate between patients with EOS and healthy controls (AUC = 0.911). Additionally, miR-34b had the highest ability to discriminate between EOS and AOS (AUC = 0.810), which may indicate different aetiological pathways to disease onset. Moreover, miR-137 dysregulation was correlated with almost all NSS subscales (i.e., sensory integration, motor sequencing, etc.) and, when EOS patients with NSS, miR-137 expression discriminated these patients from healthy controls to a greater extent (AUC = 0.957). These findings support the potential for neurodevelopmental-related miRNAs to be used as indicators of vulnerability to EOS.

## Introduction

Schizophrenia (SZ) is a severe chronic mental disorder with a wide range of symptoms characterized by neurocognitive and neurodevelopment impairments, and when a diagnosis is made during late adolescence or early adulthood, genetic predisposition plays a large role^1^. It is generally accepted that genetic predisposition to SZ is inherited, but that the disease arises after exposure to uncertain environmental factors^2^. Early-onset schizophrenia (EOS) has a stronger familial aggregation and may indicate severe long-term outcomes^3–5^, such as social disability, none living in an ordinary home^6,7^, unemployment, and even increased risk of suicide attempts^7^. Previous studies indicate that EOS and adult-onset schizophrenia (AOS) may share similar pathophysiologic features, but EOS may reflect a more severe form of the disease that is associated with a greater genetic predisposition^7,8^. Aberrant expression of molecules, such as blood-based microRNA (miRNA), may precipitate genetic and epigenetic alterations associated with the development of SZ^9,10^. The miRNAs may be associated with regulating neuronal circuit development, maturation and function through development^11^. Aberrant miRNA expression may also generate abnormal epigenetic patterns or distant regulatory elements, which may contribute to the dysregulation of critical genes involved in modulating the age of onset of SZ.

MiRNAs can be considered to be master regulators of cell-fate plasticity or determination, which may consequently be affected by disease processes^12,13^. Two large-scale genome-wide association studies (GWAS) of SZ loci implicated miR-137 enrichment as a susceptibility target for SZ association^14,15^. The largest GWAS meta-analysis of SZ to date, which systematically characterized the most important miRNAs associated with neurodevelopment and synaptic transmission, also identified miR-137 in its target gene set^16^. Furthermore, the dysregulation of miR-34a has been linked to neuronal development, which might provide critically needed insight into the pathogenesis of partial psychosis symptoms^17^. Research using induced pluripotent stem cell (iPSC)-derived neuronal cells from patients with SZ showed that neuropsychiatric disorders associated with 22q11.2 microdeletions were linked to differential expression of miR-34c, which is involved in neurological functions, including synaptogenesis, synaptic transmission, neuron development, maturation and apoptosis^18^. Only one study of EOS (*n* = 18) and healthy controls (*n* = 12) has used GWAS-derived data to demonstrate abnormal expression of miRNAs and their regulatory genes, but is limited by small sample size and requires further investigation^19^. Taken together, dynamic changes in miRNA profiles may be important epigenetic modulators linking environmental exposure to the age of onset of SZ.

The neurodevelopmental model of SZ proposes that neurological soft signs (NSS) are one of the risk factors and have potential pathophysiological significance^20,21^. NSS has also been hypothesized as markers of dysfunctional neural circuits in patients with SZ^22^. Higher NSS scores are found in unaffected first-degree relatives when compared with healthy controls, which suggests that the origins of NSS are at least partly genetic and that such abnormalities may be endophenotypes of SZ^23^. In addition, our previous study found higher overall scores of NSS in EOS compared to AOS^23^. Moreover, higher familial aggregation in the motor coordination subscale of NSS was found in EOS families than in AOS families^23^. The studies of positron emission tomography (PET) revealed that neuroinflammation across grey matter contributes to synaptic pruning in SZ-related psychosis and recent-onset patients^24–26^. Recently, a large prodromal longitudinal study of SZ also showed that miRNA expression is a potential mechanism of activated microglia by which susceptibility to SZ could alter neurodevelopment prior to disease onset^27^.

To define the genetic and/or epigenetic mechanisms underpinning the progression towards disease onset, we sought to evaluate miR-137 and the miR-34 family (i.e., miR-34a, miR-34b and miR-34c) expression patterns between patients with SZ, their unaffected first-degree relatives, and healthy controls. In addition, we included a group of patients with bipolar disorder to determine the specificity of the observed miRNA expression profiles. We established these selected miRNAs as uniquely regulated biomarkers for genetic/epigenetic susceptibility to the onset of SZ at different ages. In summary, our hypothesis is that the aberrant expression of neurodevelopment-related miRNAs may be potential biomarkers for SZ patients with NSS features and with different ages of onset. Hence, we aim to (1) compare the levels of expression of miR-137 and the miR-34 family between patients with EOS and patients with AOS; (2) evaluate the use of miRNA expression to discriminate the age of onset of SZ among patients; (3) clarify the crucial roles of miR-137 and the miR-34 family in the regulatory network of SZ.

## Results

### Descriptive data

The demographic and clinical profiles of the participants are detailed in Table 1. There were no differences in age between the patients with SZ, patients with bipolar disorder, or healthy controls. The mean age of onset for the EOS and AOS patients were well-separated (17.68 ± 3.51 and 28.99 ± 7.04 years, respectively). There were significant differences in the NSS performance scores between patients with SZ, their unaffected relatives, and healthy controls. There were no differences in gender between patients with SZ and healthy controls, but a greater proportion of males was observed among the patients with SZ than among the unaffected relatives of patients with bipolar disorder. In addition, the body mass index was different between the patients with SZ and the healthy controls. We examined the relationship between the miRNA expressions and medication use in SZ patients, the correlations were very weak and not statistically significant (Supplementary Table 1).

**Table 1:** Demographic characteristics and clinical profiles of participants.

Characteristics	Patients with schizophrenia						Unaffected first-degree relatives				Patients with bipolar disorder		Healthy controls	
	EOS (*N* = 104)		AOS (*N* = 111)		Total (*N* = 215)		REOS (*N* = 30)		RAOS (*N* = 42)		(*N* = 31)		(*N* = 100)	
	*N*	%	*N*	%	*N*	%	*N*	%	*N*	%	*N*	%	*N*	%
Male	66^a^	63.46	59	53.15	125	58.14	14	46.67	10	23.81	19	47.50	51	51.00
	**Mean**	**SD**	**Mean**	**SD**	**Mean**	**SD**	**Mean**	**SD**	**Mean**	**SD**	**Mean**	**SD**	**Mean**	**SD**
Age (years)	37.69^a,f^	11.22	44.94	8.44	41.43	10.51	56.10^d^	11.55	62.13^e^	13.65	44.95	9.22	45.56	11.44
Onset age (years)	17.68^f^	3.51	28.99	7.04	23.52	7.97	–	–	–	–	28.45	10.39	–	–
Duration (years)	20.01^f^	10.78	15.95	8.95	17.91	10.06	–	–	–	–	16.50	9.86	–	–
BMI (kg/m^2^)	25.53^a^	4.78	26.10^b^	4.84	25.83	4.81	23.04	2.98	24.46	4.32	27.20^c^	5.11	23.76	3.97
NSS	9.45^a,f^	6.99	7.80^b^	5.91	8.60	6.50	4.23^d^	3.33	5.35^e^	4.27	7.30^c^	5.50	1.33	1.78

*EOS* early-onset of schizophrenia, *AOS* adult-onset of schizophrenia, *REOS* unaffected first-degree relatives of EOS, *RAOS* unaffected first-degree relatives of AOS, *HC* healthy controls, *BMI* body mass index, *NSS* neurological soft signs.

^a^EOS vs. HC, *p* < 0.05.

^b^AOS vs. HC, *p* < 0.05.

^c^BD vs. HC, *p* < 0.05.

^d^REOS vs. HC, *p* < 0.05.

^e^RAOS vs. HC, *p* < 0.05.

^f^EOS vs. AOS, *p* < 0.05.

### MiRNA expression levels in patients with SZ and their unaffected first-degree relatives vs. psychotic and healthy controls

**Fig. 1:**
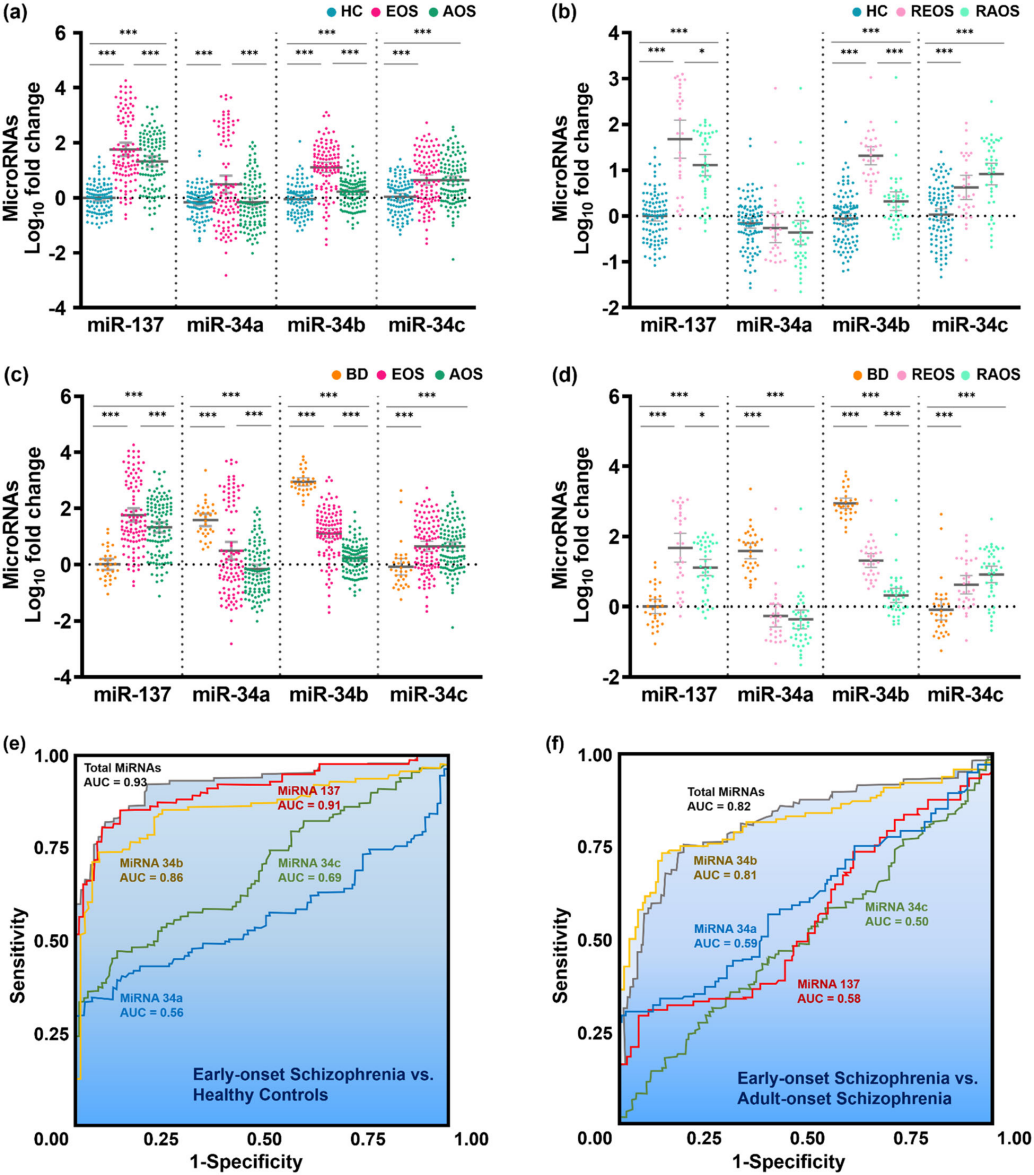
Comparison of microRNA expression levels between patients with early-onset and adult-onset schizophrenia, unaffected relatives of patients with schizophrenia, patients with bipolar disorder, and healthy controls. **a** The expression levels of the four-miRNA in the peripheral blood of EOS, AOS and HC. **b** The expression levels of the four-miRNA in the peripheral blood of REOS, RAOS and HC. **c** The expression levels of the four-miRNA in the peripheral blood of EOS, AOS and BD. **d** The expression levels of the four-miRNA in the peripheral blood of REOS, RAOS and BD. **e** Receiver operating characteristic (ROC) curve analysis of miR-137 expression levels between patients with early-onset schizophrenia and healthy controls. **f** ROC curve analysis of miR-34b expression levels between early-onset and adult-onset schizophrenia. AOS adult-onset schizophrenia, EOS early-onset schizophrenia, RAOS unaffected first-degree relatives of AOS patients, REOS unaffected first-degree relatives of EOS patients, BD bipolar disorder, HC healthy controls. *p < 0.05; **p < 0.01; ***p < 0.001. Error bars represent standard errors of the mean.

There were significant differences in the mean level of miR-137 expression (Fig. 1a), which distinguished both EOS and AOS patients from healthy controls (HC) (*p* < 0.001). On the other hand, miR-34a expression was greater in the EOS patients compared to HC (*p* < 0.001) and the AOS patients (*p* < 0.001). The expression of miR-34b distinguished the patients with EOS and AOS from the HC group, and the EOS patients from the AOS patients (*p* < 0.001). The level of miR-34c expression in HC was distinct from that of the EOS and AOS patients (*p* < 0.001). In Fig. 1b, the miR-137 and miR-34b expressions also increased in the REOS and the RAOS compared to those in HC (miR-137: REOS vs. HC, *p* < 0.001; RAOS vs. HC, *p* < 0.001. miR-34b: REOS vs. HC, *p* < 0.001; RAOS vs. HC, *p* < 0.001). Furthermore, the expressions of miR-137 and miR-34b also supported the significant results in the comparison of REOS and RAOS (*p* = 0.019 and *p* < 0.001). We further used multiple linear regression models to adjust the demographic variables (gender, age and body mass index) and the results were shown in Supplementary Tables 2–5. We also used age as a covariate in logistic regression models for adjustment and there were no obvious differences between odds ratios of unadjusted and adjusted models (Supplementary Table 6).

**Fig. 2:**
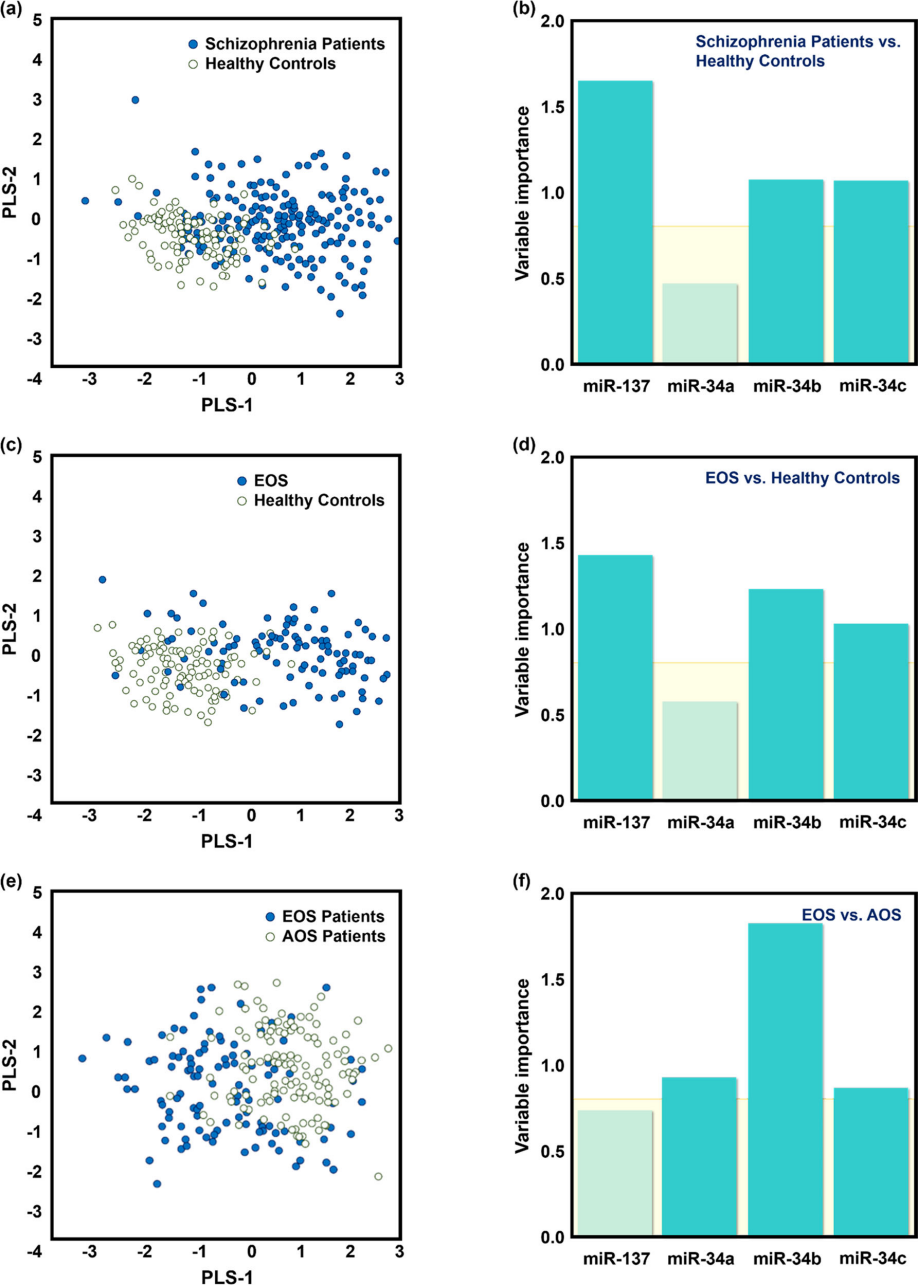
Partial least-squares discriminant analysis (PLS-DA) and variable importance in the projection (VIP) of the selected miRNAs in the study groups. Score plots derived from the PLS-DA for **a** the level of miR-137 expression between patients with schizophrenia and healthy controls, **b** the level of miR-137 expression between EOS and healthy controls, and **c** the level of miR-34b expression EOS and AOS patients. VIP data for the **d** patients with schizophrenia versus healthy controls, **e** EOS patients versus healthy controls, and **f** EOS patients versus AOS patients. AOS adult-onset schizophrenia, EOS early-onset schizophrenia.

MiR-137 was identified as a potential biomarker for the discrimination of EOS patients from the HC (AUC = 0.91) (Table 2, Fig. 1e). In addition, miR-34b could be used to distinguish EOS patients from AOS patients (AUC = 0.81, Table 2, Fig. 1f). Table 2 also shows the AUC between the unaffected first-degree relatives of EOS patients (REOS) and the unaffected first-degree relatives of AOS patients (RAOS) and the REOS and RAOS vs. HC. The correct classification accuracy and 10-fold cross-validation accuracy from the discriminant analysis between all patients with SZ, EOS patients, AOS patients, and HC are shown in Supplementary Table 7. The PLS-DA results showed that miR-137 expression improved the separation between patients with SZ and HC (Fig. 2a, b). Mir-137 and miR-34b acted as appropriate classifiers for the EOS patients vs. HC (Fig. 2c, d). Moreover, miR-34b was the best PLS factor to distinguish between EOS and AOS patients (Fig. 2e, f). The ROC curve analysis of SZ vs. patients with bipolar disorder and patients with bipolar disorder vs. HC are shown in Supplementary Table 8.

**Table 2:** ROC curve analysis of miRNA expression levels in different groups.

	miR-137	miR-34a	miR-34b	miR-34c	miR-137	miR-34a	miR-34b	miR-34c
	**SZ vs. HC**				**EOS vs. HC**			
AUC	0.885	0.517	0.751	0.699	0.911	0.567	0.862	0.693
Accuracy	0.794	0.533	0.689	0.651	0.828	0.451	0.799	0.583
Sensitivity	0.795	0.684	0.772	0.749	0.856	0.635	0.808	0.587
Specificity	0.790	0.210	0.510	0.440	0.800	0.260	0.790	0.580
	**EOS vs. AOS**				**AOS vs. HC**			
AUC	0.584	0.596	0.810	0.502	0.861	0.531	0.647	0.705
Accuracy	0.484	0.563	0.786	0.484	0.754	0.521	0.597	0.592
Sensitivity	0.404	0.423	0.740	1.000	0.802	0.991	0.685	0.622
Specificity	0.559	0.694	0.829	0.000	0.700	0.000	0.500	0.560
	**ROS vs. HC**				**REOS vs. HC**			
AUC	0.884	0.628	0.795	0.776	0.902	0.604	0.967	0.721
Accuracy	0.785	0.535	0.663	0.669	0.708	0.269	0.892	0.652
Sensitivity	0.819	0.722	0.750	0.694	0.833	0.867	0.900	0.633
Specificity	0.760	0.540	0.600	0.650	0.670	0.009	0.890	0.540
	**REOS vs. RAOS**				**RAOS vs. HC**			
AUC	0.658	0.545	0.922	0.620	0.871	0.645	0.671	0.815
Accuracy	0.667	0.333	0.833	0.625	0.732	0.373	0.542	0.683
Sensitivity	0.810	0.143	0.833	0.714	0.810	0.810	0.762	0.762
Specificity	0.467	0.600	0.833	0.500	0.700	0.019	0.450	0.650
	**SZ with NSS vs. HC**				**EOS with NSS vs. HC**			
AUC	0.927	0.662	0.742	0.690	0.957	0.598	0.826	0.749
Accuracy	0.832	0.526	0.638	0.571	0.881	0.462	0.783	0.687
Sensitivity	0.865	0.635	0.677	0.615	0.907	0.651	0.791	0.698
Specificity	0.800	0.420	0.600	0.530	0.780	0.380	0.780	0.540
	**EOS with NSS vs. AOS with NSS**				**AOS with NSS vs. HC**			
AUC	0.604	0.534	0.769	0.583	0.899	0.629	0.637	0.647
Accuracy	0.472	0.404	0.685	0.517	0.795	0.527	0.548	0.486
Sensitivity	0.558	0.535	0.767	0.674	0.826	0.695	0.630	0.630
Specificity	0.391	0.283	0.609	0.370	0.780	0.490	0.510	0.540

*AOS* adult-onset schizophrenia, *EOS* early-onset schizophrenia, *HC* healthy controls, *NSS* neurological soft signs, *RAOS* unaffected first-degree relatives of AOS patients, *ROS* unaffected first-degree relatives of patients with schizophrenia, *REOS* unaffected first-degree relatives of EOS patients, *SZ* schizophrenia.

### MiRNA expression levels in patients with SZ and NSS

The NSS scores were significantly correlated with miR-137 and miR-34a expressions in patients with SZ and NSS (Supplementary Table 9). The levels of miRNA expression in patients with SZ with and without NSS are shown in Supplementary Table 10. For miR-137, a higher AUC was obtained when comparing EOS patients with NSS to HC (AUC = 0.957), than when comparing EOS patients to HC (AUC = 0.911) (Table 2). Discriminant analysis of miRNA expression levels between the different groups of patients with SZ with NSS and HC are shown in Supplementary Table 11.

### Enrichment of functional annotation in molecular interaction is involved in the neurodevelopmental networks of patients with SZ

Bioinformatic analysis revealed the canonical pathways of miR-137 and miR-34 family involvement in the neurodevelopmental networks of patients with SZ. Few top-level pathways emerged, including L-3,4-dihydroxyphenylalanine (L-DOPA) degradation, dopamine receptor signalling, dopamine-DARPP32 (dopamine-regulated and cAMP-regulated neuronal phosphoprotein) feedback in cyclic adenosine monophosphate (cAMP) signalling, and dopamine degradation (Supplementary Fig. 1). Thorough functional enrichment analysis mapped miR-137 and the miR-34 family to disease annotations related to SZ, neurological disease and aggressive behaviour (Supplementary Table 12). The IPA^®^-Core analysis has shown the selected miRNAs and their medicated genes in Supplementary Table 13.

## Discussion

Our study examined neurodevelopment-related miRNA expression in patients with SZ with different ages of disease onset. This study was also an exploration of miRNA expression patterns among unaffected first-degree relatives (of EOS and AOS patients). MiR-137, miR-34b, and miR-34c are upregulated in patients with SZ and their unaffected first-degree relatives. In particular, among the miRNAs studied, miR-137 expression acts as the most appropriate classifier between patients with SZ and their unaffected first-degree relatives compared to healthy controls. Another unique contribution of the current study is the discovery of different miR-34b levels between EOS and AOS patients, which might indicate different aetiological pathways to disease onset. After adjusting demographic variables, the statistical significances in the expressions of miR-137 and miR-34 family between groups were similar. Hence, the major finding of this study was the identification of miR-137 and miR-34b in potentially contributing to the dysregulation of critical genes involved in determining the age of onset of SZ.

miRNAs play functional roles in genetic and epigenetic regulatory networks, including roles as ‘master regulators’, ‘switches’, or even ‘fine-tuners’ of gene expression in the context of the neurodevelopmental underpinnings of SZ^11^. MiR-137 is overexpressed in the later stages of the neurodevelopmental process, such as synaptic maturation, integration, and transmission^28,29^. In our study, the pattern of aberrant miR-137 expression discriminated patients with SZ from a control group of patients with bipolar disorder. In addition, the highly irregular expression pattern of miR-137 was specific to patients with SZ and their unaffected first-degree relatives. Several lines of evidence suggest that the expression of miR-137, and its regulation of a large number of downstream target genes, are involved in various nervous system pathways^28,29^. Numerous studies also indicated that miR-137 regulates synaptic efficacy^30^, synaptogenesis, synaptic ultrastructure, synapse function^31,32^, and its dysregulation could impair synaptic plasticity in the hippocampus^33^. These findings implicate miR-137 as one of the ‘master regulators’ in the neuropathology of SZ. The magnitude of aberrant miR-137 expression found in unaffected first-degree relatives (of patients with SZ) may further imply that miR-137 plays a dual role in the mediation of genetic risk, and epigenetic modulation of environmental exposure in the development of SZ.

Various studies have proposed that the miR-34 family may play an important role in regulating signal transduction pathways involved in neural plasticity^34,35^, regulation of stress responses^36^, stress-related psychiatric conditions^37,38^, and adverse childhood experiences or trauma^39^. Studies of patients with SZ show that miR-34a is overexpressed in early neurogenesis^40,41^, for example during the proliferation and differentiation of neural stem cells (NSCs)^17^. In our study, differences in the expression of miR-34a were observed between patients with SZ and patients with bipolar disorder, which may indicate that the pathogenesis of these diseases diverges from the time of early neurogenesis of NSCs. Previous studies have provided extensive information on the roles of miR-34b in targeting genes with established roles in autism spectrum disorder and Parkinson’s disease^42,43^ and have also shown that miR-34b is associated with common genetic variants that modulate neuroblastoma susceptibility^44^. Altered miR-34b expression discriminated between EOS and AOS in the current study, which may indicate differences in pathogenesis at a particular point in the neurodevelopmental process, and suggest that miR-34b is a ‘switch’ involved in the different aetiologies of EOS and AOS. Further, as Notch-associated miRNAs, miR-34a is important for the consolidation of fear memory^45^, miR-34b and miR-34c might be associated with depression, suicide idea and cognitive function^38^. Therefore, these findings prompted us to consider that the miR-34 family might have emerging, but still undiscovered, roles in neurodevelopment and its disorders—leaving open the possibility that observed changes in miRNA expressions might be indicators of SZ onset.

Although little is currently known about miRNA expression in first-degree relatives of patients with SZ^46^, it is evident that the relatives of these patients carry higher genetic risk and have more SZ-associated variants than healthy individuals. In the present study, we found that the levels of miR-137, miR-34b, and miR-34c were elevated both in patients with SZ and in their unaffected first-degree relatives. The similarity of altered miRNA expression patterns between patients with SZ and their unaffected first-degree relatives may play a causal role in genetic and/or epigenetic alterations. Through our functional annotation analysis for predicted target genes of the miRNAs, we gained insight into the mechanisms underlying genetic susceptibility for SZ that may be affected by the altered expression of miR-137 and the miR-34 family. Additionally, we also discovered abnormally high expression of miR-137 and miR-34b in first-degree relatives of EOS patients, rather than in first-degree relatives of AOS patients, which may reveal a phenomenon where EOS families have higher genetic or familial loading than AOS families. Recently, a cohort study of clinical high-risk young adults revealed that miRNA expression was associated with a reduction in cortical grey matter during conversion to psychosis^27^. Thus, miRNA expression is a likely mechanism by which susceptibility to psychiatric disorders could manifest and alter neurodevelopment prior to disease onset. Further studies on how miRNA expression patterns evolve may facilitate our understanding of the transition of high genetic risk individuals to psychosis or psychiatric disorders.

Aberrant early neurodevelopment is a risk factor in the trajectory of SZ, and is present long before the emergence of the clinical syndrome^46^. We discovered that miR-137 presented a higher ability to discriminate between SZ patients with NSS and HC, than patients with SZ in general vs. HC. This result led us to speculate that miR-137 might be associated with neurodevelopmental phenotypes that have sufficiently progressed to elicit functional consideration of SZ risk. Targets genes of miR-137 and the miR-34 family of particular interest were found to be involved in longitudinal processes of neurodevelopment, such as the structure and function of neurons^47^, dopamine degradation^48^, neurotransmitter release^48,49^, sensorimotor impairment^50^, and cognitive functions of SZ^50–52^. The functional aspect of bioinformatics adumbrates several neurological systems that might be influenced by the dysregulated expression of miR-137 and the miR-34 family, resulting in aberrant neurodevelopment. These findings may represent a breakthrough in the investigation of miRNA influence on the molecular aetiology of SZ.

Our findings link the altered expression of miRNAs with biological pathways that have previously been implicated in the pathogenesis of SZ; however, there are some limitations associated with the current study. First, the aberrant expression of miRNAs was observed in peripheral blood, and it remains to be confirmed whether these differences persist in the brain or in neuronal tissue. Second, we detected cell-free miRNA expression from whole blood. The different blood cells have their own distinct miRNA profiles, and they may have different proportions of blood cell subtypes between the study groups. Nevertheless, the utility of any blood-based biomarker for the diagnosis of SZ is vastly improved if it can measure in whole blood samples. Secondly, the age of SZ onset was obtained from medical records for most patients, but there was potential recall bias in the self-reported age of onset for the few patients whose medical charts were unavailable. Finally, although the miRNA expressions were not significantly associated with medication treatment in the current study, the results still need to be replicated in further studies.

Thus, our study examined the expression of neurodevelopment-related miRNAs (miR-137 and the miR-34 family) in SZ patients with different ages of SZ onset, with NSS might be presenting a unique perspective. Importantly, miR-34b has the highest discriminant ability between EOS patients and AOS patients, which might imply differences in the aetiology of SZ. Our results likely reflect the varying levels of predisposition to SZ among groups with different ages of onset and the complex heterogeneity of SZ. These intriguing findings also merit further exploration, e.g., if the expression of target genes of these miRNAs are predictive of EOS, or if differences in the expression of these genes can suggest mechanisms of resilience. It is reasonable to conclude that the biological pathways affected by the altered expression of these miRNAs are essential, which advances our mechanistic understanding of the neurodevelopmental underpinnings in genetic risk for EOS.

## Methods

### Study subjects

Patients with SZ and bipolar disorder were recruited from the Chimei Medical Center and Lok An Hospital in Taiwan from April 2011 to May 2017. We recruited subjects who met the Structured Clinical Interview for the Diagnostic and Statistical Manual for Mental Disorders, Fourth Edition (DSM-IV-TR) criteria for SZ or bipolar disorder. All recruited psychiatric patients were diagnosed by the psychiatrists from the recruited hospitals and considered to be medically stabilized. Unaffected first-degree relatives were recruited from the relatives of the proband patients with SZ who were enroled in the study. Healthy controls (HC) consisted of subjects without personal and family histories of any psychiatric disorders, recruited from hospital staff and general community. All participants were Taiwanese, of Han Chinese ethnicity, and were 20–65 years old. In total, 215 patients with SZ, 72 first-degree relatives (of the patients with SZ) without psychosis (parents, 82%; siblings, 18%), 31 patients with bipolar disorder, and 100 healthy controls were included in this study. All participants were interviewed by a well-trained research assistant with standardized psychiatric interviewing training using the Chinese version of the Diagnostic Interview for Genetic Studies (DIGS). The DIGS is a semi-structured interview that was designed specifically to assess the diagnosis of major psychiatric disorders in genetic studies^53,54^. The establishment of the Chinese version of the DIGS and its reliability has been elucidated in detail elsewhere^53^. Exclusion criteria were as follows: severe neurological abnormality, mental retardation, substance-related disorders, somatic disorder with neurological components, neurological disease or damage, significantly impaired neurocognitive function, traumatic brain injury, brain surgery, and prominent substance use. All participants gave written informed consent to be included in the study, as approved by the Institutional Review Boards (IRBs) of the participating hospitals (IRBs numbers: 10002-002, 10301-002 and B-BR-103-036-T).

### Age at onset of SZ

In our previous studies, we defined EOS as the onset of SZ in patients younger than 20 years old^23,55–58^. Thus, in the current study, all 215 patients with SZ were divided into two subgroups according to onset age: 104 EOS patients, whose onset of symptoms occurred before the age of 20; and 111 AOS patients, whose onset of symptoms occurred after the age of 20. The age of onset in the current study was determined via medical records; in the few cases where the patients’ medical charts were unavailable, the participants’ self-reported age of onset was used, or family members were consulted.

### Measurement of miRNA

Whole blood from venipuncture of a forearm vein was collected in ethylenediaminetetraacetic acid (EDTA) anticoagulant tubes for all subjects. The blood sample was separated into plasma and cellular fractions by centrifugation (800×*g*, 10 min, 4 °C) within 2 h after collection. The samples were transferred into RNase/DNase-free microcentrifuge tubes and kept at –80 °C for long-term storage.

Four individual miRNAs (miR-137, miR-34a, miR-34b, and miR-34c) were selected as follows: first, we obtained candidate genes and related miRNAs from the latest release of the DIANA-TarBase (v8.0, http://www.microrna.gr/tarbase) and miRBase (v22, http://www.mirbase.org) databases via Ingenuity^®^ Pathway Analysis (IPA^®^, QIAGEN™, Redwood City, USA) platforms. We then searched and analysed all the biological systems for miRNAs associated with neurodevelopment and SZ. Second, we identified miRNAs that were expressed in both the human brain and in peripheral blood.

Total RNA was extracted from plasma samples using TRIzol^®^ reagent (Invitrogen™, Grand Island, NY, USA) according to the manufacturer’s instructions. The purified total RNA samples (300 ng) were reverse transcribed into cDNA. Two analysis platforms were used in the current study: (1) the TaqMan^®^ Advanced miRNA Assays platform (Applied Biosystems™, Grand Island, NY, USA), for the synthesis and analysis of cDNA from input RNA; and (2) the SYBR Green assay, in which reverse transcription using the Moloney murine leukaemia virus (MMLV), and poly(A)-tailing qRT-PCR detection were performed. The sequences of the mature target miRNAs and their chromosome locations are shown in Supplementary Table 14. Each reaction was performed in triplicate, and real-time PCR data were collected by StepOne software v2.3 (Applied Biosystems™). Synthetic U-6 was used as endogenous control, and relative levels of the target miRNA in plasma were calculated based on the following formula: 2^−ΔΔCt^ (ΔΔCt = ΔCt of experimental miRNA−ΔCt of reference miRNA-U6), and the results were expressed as Log10 (2^–ΔΔCt^). Assessment of the qRT-PCR results (Supplementary Table 15) suggests that the data obtained from RNA extraction, sample storage conditions, and qRT-PCR assay in the current study were reproducible and reliable. Further, Supplementary Table 16 compares results obtained from the TaqMan^®^ and SYBR Green detection platforms and shows that both assays produce valid and reliable data.

### Assessment of NSS

NSS are subtle neurological abnormalities evaluated according to the Neurological Evaluation Scale (NES)^22^. The NSS scores were calculated from a total of 26 items, which test the following: sensory integration (e.g., finger agnosia and extinction); motor coordination (e.g., finger opposition and rapid finger tapping); motor sequencing (e.g., fist-ring, fist-edge-palm, Ozeretski test and rhythm tapping test); and others (e.g., adventitious overflow, Romberg test, tremors, testing memory 5 min/10 min, mirror movements, synkinesis, convergence, and gaze persistence). All items were scored on a three-point scale: no abnormality (0), mild but definite abnormality (1), and marked impairment (3). The standardized application of the NSS scale was established by a trained neurologist from Chimei Medical Center, who assessed and confirmed the internal consistency and inter-rater reliability of the tests. The intraclass correlation measuring the reliability of the NSS item ratings ranged from 0.77 to 1.0^23^. All participant interviews were carried out by two well-trained research assistants with experience in psychiatric wards, and who received standardized psychiatric interviewing training.

### Statistical analysis

All statistical analyses in this study were performed using SAS (SAS^®^ Institute, Cary, NC, USA, version 9.4). Two-sided *p*-values below 0.05 were considered significant. Comparisons of demographic and clinical characteristics between groups were performed as follows: (1) for categorical variables analyses was done using Pearson’s chi-square test or Fisher’s exact test; (2) for continuous variables analyses was done using the Student’s *t*-test, or a one-way analysis of variance (ANOVA) with Tukey’s multiple comparisons test to detect pairwise differences between groups. The Pearson’s correlation coefficients were used to investigate the relationship between miRNA expression and NSS subscales. The receiver-operating characteristic (ROC) curve in logistic regression models were used to evaluate the discriminant ability of miRNA expression in the study groups. We also estimated the correct classification accuracy and the 10-fold cross-validation accuracy of miRNA expression between groups. The variable importance in projection (VIP) scores used the partial least-squares discriminant analysis (PLS-DA) between groups to estimate the importance of each miRNA.

## Supplementary information

Supplementary Information

## Data Availability

The datasets used and analysed in the current study are not publicly available due to conditions on participant consent and other ethical restrictions.

## Code availability

The SAS code that supports the findings of this study are available from the corresponding author upon reasonable request.
