# Supplementary material for: Neurodevelopment regulators miR-137 and miR-34 family as biomarkers for early and adult onset schizophrenia
Source: NPJ Schizophr. 2021 Jul 5;7:35. doi: 10.1038/s41537-021-00164-1 (PMC8257739; doi:10.1038/s41537-021-00164-1)
Supplement: Supplementary file 1 — Supplementary Information [file 41537_2021_164_MOESM1_ESM.pdf]

Supplementary Table 1. Correlations between miRNA expression levels and demographic variables of schizophrenia patients.

	miR-137			miR-34a			miR-34b			miR-34c		
Variables	r	95% CI	P-value	r	95% CI	P-value	r	95% CI	P-value	r	95% CI	P-value
Gender [a]	0.10	-0.03 - 0.24	0.13	-0.06	-0.19 - 0.08	0.42	0.04	-0.09 - 0.17	0.55	0.10	-0.30 - 0.23	0.13
Age (year)	-0.19	-0.31 - -0.05	0.01	0.22	0.09 - 0.35	< 0.01	-0.23	-0.35 - -0.10	< 0.01	-0.16	-0.29 - -0.27	0.02
BMI (kg/m^2)	0.06	-0.07 - 0.20	0.35	-0.22	-0.16 - 0.11	0.78	0.10	-0.04 - 0.23	0.16	0.09	-0.05 - 0.22	0.21
Antipsychotics	-0.12	-0.94 - 0.17	0.40	-0.11	-0.38 - 0.18	0.46	0.07	-0.22 - 0.35	0.63	-0.09	-0.36 - 0.20	0.56
Sedatives / Hypnotics / Anxiolytics	0.18	-0.17 - 0.48	0.32	0.36	0.02 - 0.61	0.04	0.23	-0.11 - 0.53	0.18	0.05	-0.29 - 0.38	0.77

The correlations between miRNA expressions and variables of schizophrenia patients by Spearman's correlation or point-biserial correlation [a]. BMI, body mass index; CI: confidence interval. Antipsychotic treatment was converted to oral chlorpromazine equivalent dose (mg/day), sedatives / hypnotics / anxiolytics treatment was converted to fluphenazine equivalents (mg/day) [1].

Supplementary Table 2. Multiple linear regression analysis with miRNA expression levels as the dependent variable between EOS, AOS and HC.

	miR-137			miR-34a			miR-34b			miR-34c		
Variables	β	95% CI	P-value	β	95% CI	P-value	β	95% CI	P-value	β	95% CI	P-value
**Model 1**												
EOS vs. HC	1.54	1.24 - 1.84	< 0.01	0.60	0.26 - 0.93	< 0.01	1.02	0.80 - 1.25	< 0.01	0.48	0.34 - 0.73	< 0.01
AOS vs. HC	1.30	1.03 - 1.57	< 0.01	0.25	-0.05 - 0.56	0.11	0.34	0.14 - 0.54	< 0.01	0.60	0.35 - 0.79	< 0.01
**Model 2**												
EOS vs. AOS	0.24	-0.04 - 0.52	0.09	0.34	0.03 - 0.66	0.03	0.68	0.47 - 0.89	< 0.01	-0.09	-0.32 - 0.15	0.46
HC vs. AOS	-1.30	-1.57 - -1.03	< 0.01	-0.25	-0.56 - 0.05	0.11	-0.34	-0.54 - -0.14	< 0.01	-0.60	-0.79 - -0.35	< 0.01

EOS: early-onset of schizophrenia; AOS: adult-onset of schizophrenia; HC: healthy controls. Model 1: HC as the reference group for comparing groups; Model 2: AOS as the reference group for comparing groups. The demographic variables (gender, age and BMI) were considered as the covariates for the multiple linear regression.

Supplementary Table 3. Multiple linear regression analysis with miRNA expression levels as the dependent variable between REOS, RAOS and HC.

	miR-137			miR-34a			miR-34b			miR-34c		
Variables	β	95% CI	P-value	β	95% CI	P-value	β	95% CI	P-value	β	95% CI	P-value
**Model 1**												
REOS vs. HC	1.68	1.37 - 1.99	< 0.01	-0.17	-0.47 - 0.13	0.26	1.36	1.11 - 1.61	< 0.01	0.54	0.25 - 0.84	< 0.01
RAOS vs. HC	1.12	0.81 - 1.43	< 0.01	-0.23	-0.53 - 0.06	0.12	0.41	0.16 - 0.66	< 0.01	0.82	0.53 - 1.12	< 0.01
**Model 2**												
REOS vs. RAOS	0.56	0.21 - 0.92	< 0.01	0.07	-0.27 - 0.40	0.70	0.95	0.66 - 1.24	< 0.01	0.28	-0.61 - 0.05	0.10
HC vs. RAOS	-1.12	-1.43 - -0.81	< 0.01	0.23	-0.06 - 0.53	0.12	-0.41	-0.66 - -0.16	< 0.01	-0.82	-1.12 - -0.53	< 0.01

REOS: nonpsychotic first-degree relatives of EOS; RAOS: nonpsychotic first-degree relatives of AOS; HC: healthy controls. Model 1: HC as the reference group; Model 2: RAOS as the reference group. The demographic variables (gender, age and BMI) were considered as the covariates for the multiple linear regression.

Supplementary Table 4. Multiple linear regression analysis with miRNA expression levels as the dependent variable between EOS, AOS and BD.

	miR-137			miR-34a			miR-34b			miR-34c		
Variables	β	95% CI	P-value	β	95% CI	P-value	β	95% CI	P-value	β	95% CI	P-value
EOS vs. BD	1.60	1.13 - 2.07	< 0.01	-0.69	-1.28 - -0.10	0.02	-1.78	-2.14 - -1.43	< 0.01	0.65	0.27 - 1.03	< 0.01
AOS vs. BD	1.32	0.95 - 1.69	< 0.01	-1.74	-2.06 - -1.41	< 0.01	-2.72	-2.93 - -2.50	< 0.01	0.71	0.38 - 1.05	< 0.01

EOS: early-onset of schizophrenia; AOS: adult-onset of schizophrenia; BD: bipolar disorder. The demographic variables (gender, age and BMI) were considered as the covariates for the multiple linear regression.

Supplementary Table 5. Multiple linear regression analysis with miRNA expression levels as the dependent variable between REOS, RAOS and BD.

	miR-137			miR-34a			miR-34b			miR-34c		
Variables	β	95% CI	P-value	β	95% CI	P-value	β	95% CI	P-value	β	95% CI	P-value
REOS vs. BD	1.67	1.11 - 2.23	< 0.01	-1.99	-2.46 - -1.52	< 0.01	-1.70	-1.99 - -1.41	< 0.01	0.52	0.05 - 1.00	0.03
RAOS vs. BD	1.21	0.81 - 1.62	< 0.01	-2.11	-2.57 - -1.64	< 0.01	-2.57	-2.90 - -2.44	< 0.01	1.14	0.67 - 1.61	< 0.01

REOS: nonpsychotic first-degree relatives of EOS; RAOS: nonpsychotic first-degree relatives of AOS; BD: bipolar disorder. The demographic variables (gender, age and BMI) were considered as the covariates for the multiple linear regression.

Supplementary Table 6. Age-adjusted odds ratios for microRNA expression associated with study groups

	SZ vs. HC			***EOS vs. HC***			***EOS vs. AOS***		
MicroRNA	Odds ratio	95% CI	P-value	Odds ratio	95% CI	P-value	Odds ratio	95% CI	P-value
miR-137	8.09	4.91 - 13.34	< 0.01	12.84	6.25 - 26.40	< 0.01	1.41	1.10 - 1.80	0.01
(Age adj.)	7.39	5.12 - 12.85	< 0.01	11.30	6.56 - 24.71	< 0.01	1.35	1.11 - 1.72	0.02
miR-34a	1.23	1.03 - 1.61	0.03	1.58	1.23 - 2.03	0.04	1.48	1.19 - 1.84	< 0.01
(Age adj.)	1.18	1.12 - 1.58	0.05	1.49	1.35 - 1.90	0.07	1.39	1.25 - 1.69	< 0.01
miR-34b	3.47	2.36 - 5.11	< 0.01	6.59	3.95 - 11.00	< 0.01	4.78	2.99 - 7.65	< 0.01
(Age adj.)	3.09	2.57 - 4.88	< 0.01	6.01	4.03 - 10.77	< 0.01	3.99	3.02 - 7.26	< 0.01
miR-34c	2.50	1.80 - 3.47	< 0.01	2.54	1.73 - 3.71	< 0.01	1.01	0.75 - 1.36	0.96
(Age adj.)	2.45	1.80 - 3.39	< 0.01	2.49	1.85 - 3.69	< 0.01	1.00	0.76 - 1.29	0.99

SZ, schizophrenia; AOS, adult-onset schizophrenia; BMI, body mass index; EOS, early-onset schizophrenia; CI: confidence interval; Adj.: adjustment

Supplementary Table 7. Discriminant analysis of miRNA expression levels in different groups.

	SZ vs. HC				***EOS vs. HC***			
Variables	miR-137	miR-34a	miR-34b	miR-34c	miR-137	miR-34a	miR-34b	miR-34c
Correct classification accuracy (%)	80.32	66.03	69.20	67.62	86.76	70.10	81.37	65.20
Cross-validation accuracy (%)	80.00	65.40	68.25	66.03	86.76	70.10	81.37	64.71
	EOS vs. AOS				***AOS vs. HC***			
Correct classification accuracy (%)	54.88	60.93	80.00	51.63	79.62	63.03	60.91	63.51
Cross-validation accuracy (%)	54.88	60.93	79.53	51.16	79.62	63.03	60.91	63.51

SZ, schizophrenia; EOS, early-onset schizophrenia; AOS, adult-onset schizophrenia; HC, healthy controls.

Supplementary Table 8. ROC curve analysis of miRNA expression levels in different groups.

	SZ vs. BD				***BD vs. HC***			
	miR-137	miR-34a	miR-34b	miR-34c	miR-137	miR-34a	miR-34b	miR-34c
AUC	0.885	0.828	0.988	0.750	0.506	0.988	1.000	0.599
Accuracy	0.821	0.790	0.972	0.762	0.730	0.942	0.993	0.358
Sensitivity	0.837	0.819	0.981	0.791	0.000	0.973	1.000	0.892
Specificity	0.730	0.622	0.919	0.595	1.000	0.930	0.990	0.160

ROC, receiver operating characteristic; SZ, schizophrenia; BD, bipolar disorder; HC, healthy controls.

Supplementary Table 9. Significance of correlation between miRNA expression levels and NSS scores in schizophrenia patients.

	NSS Subscales				
MicroRNA (*Log_{10} fold change*)	*Sensory integration*	*Motor coordination*	*Motor sequencing*	*Other*	*Total NSS score*
miR-137	0.2435***	0.0705	0.2147***	0.1588*	0.2270***
miR-34a	0.1213	0.0274	0.1772**	0.2112**	0.1953**
miR-34b	0.0550	-0.0356	0.0262	0.0121	0.0243
miR-34c	0.0415	-0.0305	-0.0132	0.0311	0.0170

NSS, neurological soft signs. * $p < 0.05$; ** $p < 0.01$; *** $p < 0.001$.

Supplementary Table 10. Comparison of miRNA expression levels in patients with schizophrenia with and without NSS.

	With NSS (N = 96)	Without NSS (N = 119)	
MicroRNA *(Log$_{10}$ fold change)*	Mean (SD)	Mean (SD)	P-value
miR-137	1.77 (1.12)	1.34 (1.13)	0.0061
miR-34a	0.42 (1.34)	-0.06 (1.29)	0.0085
miR-34b	0.65 (0.88)	0.66 (0.88)	0.9628
miR-34c	0.64 (1.02)	0.64 (0.80)	0.9813

NSS, neurological soft signs.

Supplementary Table 11. Discriminant analysis of miRNA expression levels in different groups.

	SZ with NSS vs. HC				
Variables	Total	miR-137	miR-34a	miR-34b	miR-34c
Correct classification accuracy (%)	92.86	87.56	68.88	68.88	71.43
Cross-validation accuracy (%)	90.31	87.56	68.37	66.84	70.92
	EOS with NSS vs. HC				
Correct classification accuracy (%)	95.80	89.51	78.32	86.01	81.82
Cross-validation accuracy (%)	94.41	89.51	78.32	86.01	81.82
	AOS with NSS vs. HC				
Correct classification accuracy (%)	92.47	86.99	73.97	86.49	76.71
Cross-validation accuracy (%)	91.78	86.99	73.97	86.49	76.71

SZ, schizophrenia; EOS, early-onset schizophrenia; AOS, adult-onset schizophrenia; NSS, neurological soft signs; HC, healthy controls

Supplementary Table 12. Top five diseases and functional annotations of selected miRNAs based on IPA®-Core Analysis.

Categories	Disease or functional annotation	P-value	Number of molecules	Molecules
Neurological disease, psychological disorders	**Schizophrenia**	9.19E-09	8	CACNA1C, COMT, DRD2, **mir-137**, **miR-34a-5p** (and other miRNAs w/seed GGCAGUG), PTGS2, TCF4, ZNF804A
Cancer, neurological disease, organismal injury and abnormalities	Brain cancer	7.32E-08	6	CDK6, EZH2, KDM1A, MIB1, PTGS2, TCF4
Neurological disease, organismal injury and abnormalities	Brain lesion	1.18E-07	9	CACNA1C, CDK6, DRD2, EZH2, KDM1A, MIB1, PTGS2, TCF4, ZNF804A
Embryonic development, organismal development, tissue development	Growth of embryonic tissue	1.92E-07	6	CDK6, DRD2, KDM1A, NR2E1, PTGS2, TCF4
Behaviour	Aggressive behaviour	3.22E-07	4	COMT, DRD2, NR2E1, PTGS2

IPA®: Ingenuity® Pathway Analysis. Diseases or molecules names shown in bold type were investigated in the current study.

Supplementary Table 13. Overall molecular biological characteristics, annotations, and applications of selected miRNA-medicated gene as drugs target based on IPA®-Core Analysis.

Symbol	Entrez Gene [a] name	Human ID	Location	Type	Biomarker applications	Drugs
CDK6	Cyclin dependent kinase 6	1021	Nucleus	Kinase	-	Palbociclib, ribociclib, abemaciclib, letrozole/palbociclib, FLX925, fulvestrant/palbociclib, trilaciclib, G1T38, letrozole/ribociclib, anastrozole/palbociclib, anastrozole/ribociclib, exemestane/palbociclib, exemestane/ribociclib, alvocidib
EZH2	Enhancer of zeste 2 polycomb repressive complex 2 subunit	2146	Nucleus	Transcription regulator	Diagnosis, disease progression, efficacy	tazemetostat, GSK2816126, CPI-1205, DS-3201
TLX1	T cell leukaemia homeobox 1	3195	Nucleus	Transcription regulator	-	-
TCF4	Transcription factor 4	6925	Nucleus	Transcription regulator	-	-
KDM1A	Lysine demethylase 1A	23028	Nucleus	Enzyme	Unspecified application	GSK-2879552, RO7051790, IMG-7289, CC-90011, INCB059872
miR-34a-3p	-	-	Cytoplasm	Mature miRNA	-	-
miR-34a-5p	-	-	Cytoplasm	Mature miRNA	Unspecified application	-
miR-34b-3p	-	-	Cytoplasm	Mature miRNA	Unspecified application	-
miR-34c-3p	-	-	Cytoplasm	Mature miRNA	-	-
COMT	Catechol-O-methyltransferase	1312	Cytoplasm	Enzyme	Diagnosis, efficacy, prognosis, unspecified application	Carbidopa/entacapone/levodopa, BIA-3-202, tolcapone, entacapone
PTGS2	Prostaglandin-endoperoxide synthase 2	5743	Cytoplasm	Enzyme	Diagnosis, disease progression, efficacy, prognosis, unspecified application	Acetaminophen/pentazocine, acetaminophen/clemastine/pseudoephedrine, aspirin/butalbital/caffeine, acetaminophen/caffeine/dihydrocodeine, aspirin/hydrocodone, aspirin/oxycodone, acetaminophen/aspirin/caffeine, aspirin/pravastatin, acetaminophen/dexbrompheniramine/pseudoephedrine,

Symbol	Entrez Gene [a] name	Human ID	Location	Type	Biomarker applications	Drugs
PTGS2 *(continued)*	Prostaglandin-endoperoxide synthase 2	5743	Cytoplasm	Enzyme	Diagnosis, disease progression, efficacy, prognosis, unspecified application	aspirin/meprobamate, aspirin/caffeine/propoxyphene, aspirin/butalbital/caffeine/codeine, aspirin/caffeine/dihydrocodeine, chlorpheniramine/ibuprofen/pseudoephedrine, licofelone, menatetrenone, icosapent, suprofen, lornoxicam, tiaprofenic acid, lumiracoxib, tenoxicam, naproxen/sumatriptan, apricoxib, parecoxib, ibuprofen/phenylephrine, acetaminophen/aspirin/codeine, esomeprazole/naproxen, aspirin/esomeprazole, aspirin/dipyridamole/telmisartan, famotidine/ibuprofen, aspirin/dabigatran etexilate, diclofenac/omeprazole, chlorpheniramine/ibuprofen/phenylephrine, dexamethasone/pomalidomide, sulindac/tamoxifen, sulindac/toremifene, raloxifene/sulindac, ketorolac/phenylephrine, Aspirin/bivalirudin, diclofenac/hyaluronic acid, aspirin/clopidogrel, aspirin/omeprazole, aspirin/enoxaparin, aspirin/lisinopril, COX2 inhibitor, diclofenac/misoprostol, acetaminophen/butalbital/caffeine, hydrocodone/ibuprofen, acetaminophen/hydrocodone, acetaminophen/tramadol, acetaminophen/codeine, acetaminophen/oxycodone, acetaminophen/propoxyphene, niflumic acid, nitroaspirin, ketoprofen, diclofenac, etoricoxib, naproxen, meclofenamic acid, pomalidomide, meloxicam, celecoxib, ibuprofen/pseudoephedrine, diphenhydramine/ibuprofen, dipyrone, nimesulide, acetaminophen, mefenamic acid, bortezomib/dexamethasone/pomalidomide, diflunisal, ibuprofen, GW406381X, phenylbutazone, indomethacin, sulfasalazine, piroxicam, valdecoxib, aspirin, carprofen, zomepirac, rofecoxib, sorafenib/sulindac/sunitinib, aspirin/caffeine/orphenadrine, acetaminophen/butalbital, balsalazide, aspirin/dipyridamole, acetaminophen/butalbital/caffeine/codeine, naproxen/pseudoephedrine, acetaminophen/diphenhydramine/prednisolone, acetaminophen/diphenhydramine/methylprednisolone, acetaminophen/diphenhydramine, acetaminophen/cetirizine/prednisolone, racemic flurbiprofen, phenacetin, sulindac, nabumetone, etodolac, tolmetin, ketorolac, oxaprozin, mesalamine, salsalate, fenoprofen, salicylic acid, Acetaminophen/caffeine/chlorpheniramine/hydrocodone/phenylephrin-e, bromfenac
MIB1	Mindbomb E3 ubiquitin protein ligase 1	57534	Cytoplasm	Enzyme	diagnosis, efficacy	-
miR-137	MiRNA 137	406928	Cytoplasm	MiRNA	-	-

Symbol	Entrez Gene [a] name	Human ID	Location	Type	Biomarker applications	Drugs
CACNA1C	Calcium voltage-gated channel subunit alpha1 C	775	Plasma membrane	Ion channel	-	Cinnarizine, MEM-1003, amlodipine/olmesartan medoxomil, clevidipine butyrate, amlodipine/hydrochlorothiazide/valsartan, amlodipine/telmisartan, aliskiren/amlodipine, amlodipine/hydrochlorothiazide/olmesartan medoxomil, aliskiren/amlodipine/hydrochlorothiazide, nilvadipine, amlodipine/valsartan, amlodipine/perindopril, amlodipine/benazepril, diltiazem, verapamil, dexamethasone/rituximab/verapamil, mibefradil, bepridil, enalapril/felodipine, amlodipine/atorvastatin, nisoldipine, isradipine, felodipine, nimodipine, nitrendipine, amlodipine, nicardipine, nifedipine, ibutilide, trandolapril/verapamil, diltiazem/enalapril
DRD2	Dopamine receptor D2	1813	Plasma membrane	G-protein coupled receptor	Response to therapy	Paliperidone, **risperidone**, buspirone, carbidopa/entacapone/levodopa, bifeprunox, iloperidone, blonanserin, asenapine, pardoprunox, ocaperidone, abaperidone, methotrimeprazine, fluspirilene, SLV-314, cariprazine, rotigotine, acetophenazine, BIM 23A760, sultopride, zuclopenthixol, thioproperazine, lurasidone, opipramol, paliperidone palmitate, brexpiprazole, pipothiazine, benperidol, carbidopa/levodopa, **chloropromazine**, **domperidone**, metoclopramide, sulpiride, meloxicam, amantadine, flupenthixol, chlorprothixene, trifluoperazine, dexamethasone/olanzapine, haloperidol/octreotide, fluphenazine, pimozide, **clozapine**, haloperidol, fluoxetine/olanzapine, fluphenazine decanoate, thiothixene, amitriptyline/perphenazine, haloperidol decanoate, molindone, trimethobenzamide, fluphenazine enanthate, loxapine, perphenazine, promazine, prochlorperazine, triflupromazine, quetiapine, pramipexole, olanzapine, remoxipride, lisuride, sertindole, cabergoline, **ziprasidone**, mesoridazine, thioridazine, **aripiprazole**, ropinirole, dihydroergocryptine, dihydroergotamine, bromocriptine, apomorphine, pergolide, dopamine, droperidol, thiethylperazine, droperidol/fentanyl, L-dopa
ZNF804A	Zinc finger protein 804A	91752	Plasma membrane	Other	-	-

IPA®: Ingenuity® Pathway Analysis. Drugs shown in bold type indicate antipsychotic medications. [a] Entrez Gene (*http://www.ncbi.nlm.nih.gov/gene*) is the National Center for Biotechnology Information (NCBI)'s database for gene-specific information, which maintains records from genomes that have been completely sequenced, have an active research community to submit gene-specific information, or are scheduled for intense sequence analysis.

Supplementary Table 14. List of primer sequences for mature miRNA tested in the current study.

miRNA ID	Chromosome location	Species	Mature miRNA sequence
hsa-miR-137	Chr.1: 98046070–98046171 [–] on build GRCh38	*Human*	UUAUUGCUUAAGAAUACGCGUAG
hsa-miR-34a	Chr.1: 9151668–9151777 [–] on build GRCh38	*Human*	UGGCAGUGUCUUAGCUGGUUGU
hsa-miR-34b	Chr.11: 111512938–111513021 [+] on build GRCh38	*Human, Pan troglodytes, Pongo pygmaeus, Rhesus monkey*	CAAUCACUAACUCCACUGCCAU
hsa-miR-34c	Chr.11: 111513439–111513515 [+] on build GRCh38	*Human*	AGGCAGUGUAGUUAGCUGAUUGC

ID, identification; miRNA, microRNA.

Supplementary Table 15. The reproducibility of the quantitative reverse transcription polymerase chain reaction assay.

	Time point (day)							
MicroRNA	1	2	3	4	5	Mean	SD	CV (%)
miR-137 (Ct)	26.81	26.95	26.76	26.06	27.01	26.72	0.38	1.4
miR-34a (Ct)	32.65	32.56	32.22	32.12	33.11	32.53	0.39	1.2
miR-34b (Ct)	29.32	29.48	29.50	28.43	27.58	28.87	0.84	2.9
miR-34c (Ct)	30.03	29.08	30.23	29.33	30.80	29.89	0.70	2.3

Ct, comparative threshold; CV, coefficient of variation.

Supplementary Table 16. Comparison of miRNA expression levels in EOS and AOS patients, quantified by SYBR Green assays vs. TaqMan assays.

	EOS (N = 30)			AOS (N = 30)		
	SYBR Green	TaqMan		SYBR Green	TaqMan	
MicroRNA *(Log$_{10}$ fold change)*	Mean (SD)	Mean (SD)	P-value	Mean (SD)	Mean (SD)	P-value
miR-137	1.10 (1.17)	1.06 (1.16)	0.8895	0.37 (0.57)	0.33 (0.57)	0.8072
miR-34a	–0.41 (0.80)	–0.43 (0.78)	0.9081	–0.88 (0.46)	–0.79 (0.43)	0.4557

EOS, early-onset schizophrenia; AOS, adult-onset schizophrenia.

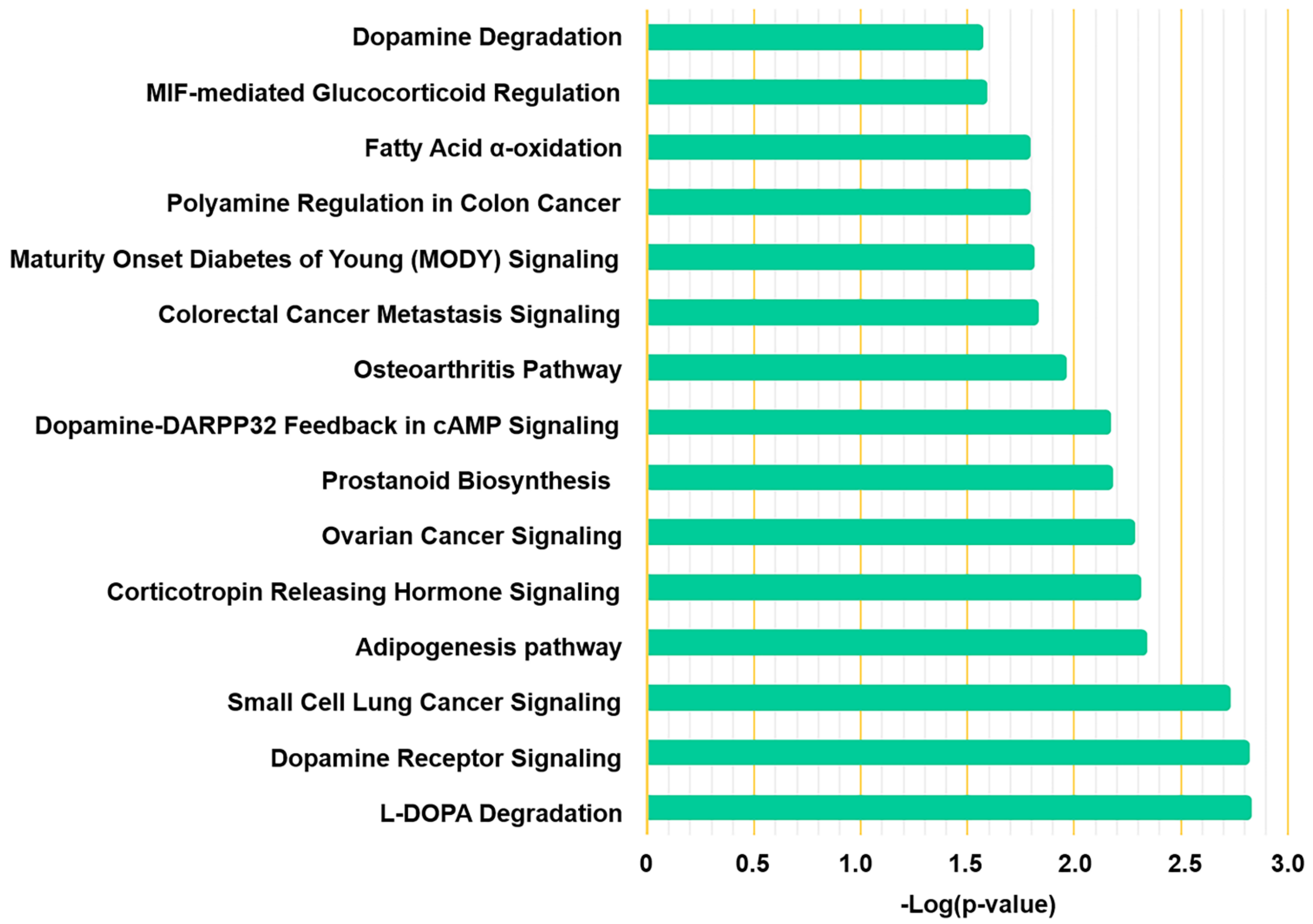

Supplementary Fig. 1 Top canonical pathways associated with miR-137 and the miR-34 family in patients with schizophrenia.

cAMP, cyclic adenosine monophosphate; dopamine-DARPP32, dopamine- and cAMP-regulated neuronal phosphoprotein; MIF, macrophage migration inhibitory factor.

Supplementary Table References

1 Inada, T. & Inagaki, A. Psychotropic dose equivalence in Japan. *Psychiatry Clin. Neurosci.* **69**, 440-447 (2015).
